# Dosimetric evaluation of the feasibility of utilizing a reduced number of interstitial needles in combined intracavitary and interstitial brachytherapy for cervical cancer

**DOI:** 10.1002/acm2.13833

**Published:** 2022-11-10

**Authors:** Dishary Jahan, Salahuddin Ahmad, Spencer Thompson, Erich Schnell

**Affiliations:** ^1^ Departments of Radiation Oncology and Radiological Sciences The University of Oklahoma Health Sciences Center Oklahoma City Oklahoma USA

**Keywords:** brachytherapy, cervical carcinoma, combined interstitial/intracavitary, high dose rate, Venezia

## Abstract

**Purpose:**

To evaluate the ability of the Venezia advanced multichannel tandem and ring applicator to consistently produce dosimetrically comparable plans utilizing a reduced number of needle channels, to reduce the risk of secondary complications when boosting cervical cancer treatments with high dose rate (HDR) brachytherapy.

**Methods:**

We evaluated 26 fractions from 13 patients who were treated with HDR brachytherapy using the Venezia (Elekta) applicator. The original plans included a full load of 12–16 needles, including both parallel and 30‐degree oblique needles. We replanned each original to nine new configurations, with a reduced number of two, three, four, or six needles. Comparisons included differences in percentage dose coverage to 90% of the high‐risk clinical target volume, and percentage dose to 2 cm^3^ of the bladder, rectum, sigmoid, and bowel. We considered new plans “passing” if they remained within our standards (*D*90 > 100%; *D*2 cm^3^ < 85% bladder, <65% rectum, sigmoid, bowel) or did not perform worse than original.

**Results:**

Removing only the two most anterior or the two most posterior needles from both sides showed 80.8% and 61.5% overall passing rate. Removal of the most anterior and posterior four needles together showed 65.4% overall passing rate. Removing all oblique needles showed 19.2% overall passing rate. Removing only left‐sided or only right‐sided oblique needles showed 46.2% and 23.1% overall passing, respectively. Removing only right‐sided or only left‐sided parallel needles separately showed 19.2% and 34.6% overall passing, respectively. Removing all parallel needles showed 11.5% overall passing rate.

**Conclusions:**

As only two replans required a full needle load to maintain dosimetric quality and 40 (76.9%), 36 (34.6%), 18 (69.2%), and 10 (19.2%) replans passed with 2, 3, 4, and 6 needles removed respectively, this indicates the potential for using a lesser number of interstitial needles during combined intracavitary and interstitial HDR brachytherapy while maintaining dosimetric quality.

## 1 INTRODUCTION

Cervical cancer is the fourth most widespread cancer in women worldwide.^1^ According to the National Cancer Institute, 14480 new cases of cervical cancer have been estimated as of 2021, with 4290 deaths in the United States.^2^ In 2018, an estimated 570000 people worldwide were diagnosed with cervical cancer and about 311000 women died of the disease, according to the World Health Organization.^3^

A standard radiation treatment for locally advanced cervical cancer is external beam radiation therapy (EBRT) with concurrent *cis*‐platinum‐based chemotherapy followed by brachytherapy.^4^, ^5^, ^6^, ^7^, ^8^, ^9^ Brachytherapy is an important part of the treatment process that has been linked to better cancer‐specific and overall survival.^10^ Boosting dose with brachytherapy after the EBRT provided a 12% better overall survival rate than treatment with only EBRT.^11^ Cervical cancer can be treated with several types of different applicators, either intracavitary or interstitial. With a tandem and ring intracavitary applicator alone, it can be difficult to deliver a sufficient dose to the high‐risk clinical target volume (HRCTV) in patients with bulky disease or parametrial extension.^12^, ^13^, ^14^ Therefore, the addition of interstitial needles with the intracavitary applicator results in target dose escalation while respecting the OAR limits.^15^

**FIGURE 1:**
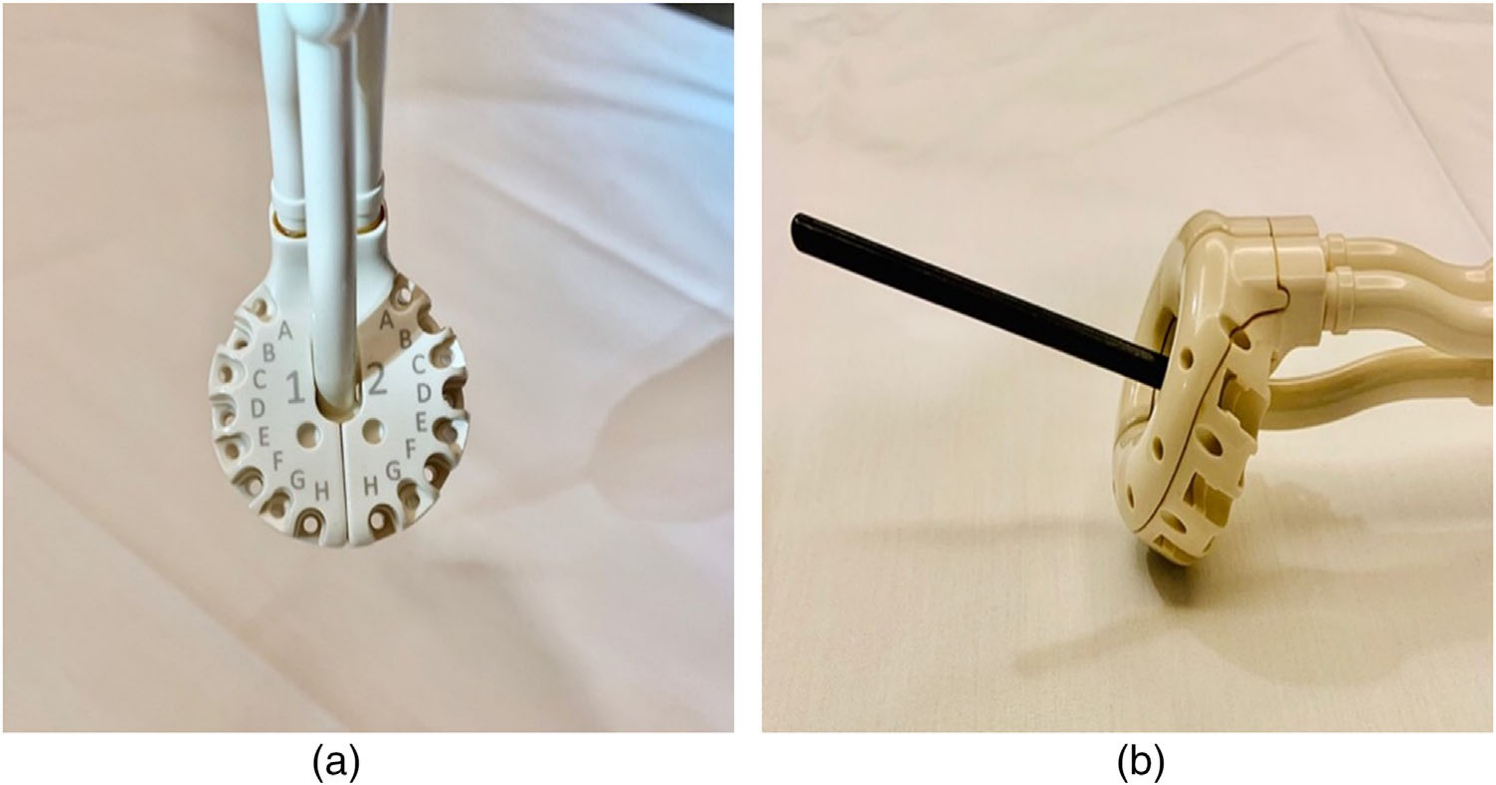
Venezia advanced multichannel applicator (a) and with tandem (black rod) (b) (where “1” and “2” indicate left and right sides of the applicator, respectively)

**FIGURE 2:**
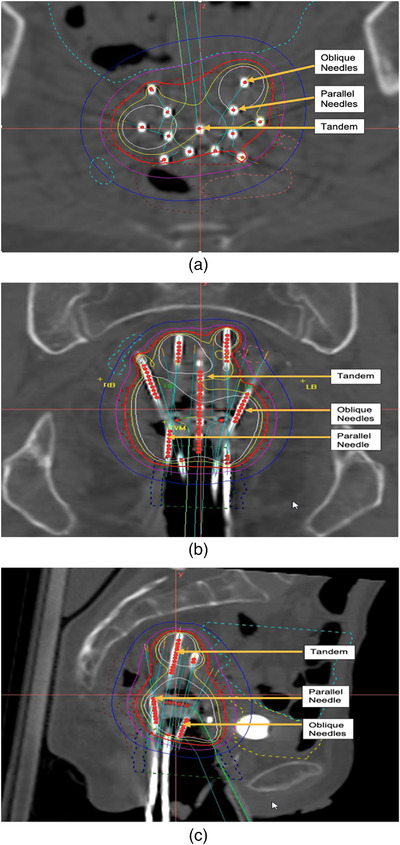
(a) Axial, (b) coronal, and (c) sagittal, CT view of Venezia applicator with needle orientation during treatment showing dose distribution by tandem, parallel and oblique needles

This research deals specifically with combined Intracavitary and interstitial brachytherapy using an advanced gynecological hybrid applicator Venezia (Elekta, Sweden) to treat the targets larger than the standard dose distribution (≥30 cm^3^) for tandem and ring alone. This advanced applicator combined intracavitary and interstitial high dose rate (HDR) brachytherapy through the use of an interstitial ring to improve dose distribution to the cancers that spread into the parametrium and vagina. The Venezia applicator is shown in Figure 1 and consists of two lunar‐shaped ovoids that snap together to form a ring that includes 12–16 needle channels, depending on the diameter. Needles for the purpose of brachytherapy are hollow plastic channels with a sharp tip, placed using a solid removable metal core. The lunar ovoid holes enable the placement of channels parallel, or oblique (30 degrees) to the tandem. A study by Walter et al.^16^ found that the Venezia applicator's clinical implementation is feasible and allows for substantially increased dose coverage while still sparing organs at risk. The Venezia is compatible with all imaging modalities and is visible in almost every imaging workflow, including ultrasound, CT, X‐ray, and MRI. Figure 2 shows the axial, coronal, and sagittal, CT views of the Venezia applicator with needles during treatment.

Interstitial needles improve the dose optimization during the treatment, but HDR brachytherapy is usually a fractionated treatment procedure where the applicator needs to be placed in the patient before each fraction. This can cause increased trauma and pain to the patient due to repeated puncture when using interstitial needles. Using a greater number of needles can increase the secondary deleterious effect by increasing the number of perforations the normal tissue or organs receive during each fraction. Puncturing critical organs of bladder, rectum, sigmoid, and bowel can cause toxicity in addition to bleeding, which is the most common consequence of the insertion of needles. It can increase the chance of all levels of acute toxicity.

The hypothesis of this study is that it is possible to create plans of an equal quality to the original clinical plans that utilized all needle channels available in the interstitial ring, while utilizing fewer needles. Doing so will thereby lowering the possibility of secondary complications when using combined intracavitary and interstitial HDR brachytherapy. If achievable, this will help reduce the patient detriment and secondary toxicity due to the use of interstitial needles during the treatment of cervical cancer.

## 2 MATERIALS AND METHODS

### 2.1 Patients characteristics

The dosimetric evaluations were performed on 26 brachytherapy fractions from 13 patients with cervical cancer, who were treated with the Venezia multichannel tandem and ring applicator in HDR brachytherapy. HDR brachytherapy was given to all patients as a boost after the external beam radiation and chemotherapy. The dose for each fraction was 600 cGy, with five fractions constituting the standard course of treatment. General anesthesia was given to the patients during the insertion of interstitial needles for their therapy. The average treatment time ranged from 3 to 5 h per patient with specialized multichannel applicators. The median age of all patients at the time of the treatment was 47 years, ranging from 26 to 78 years. Patient characteristics are shown in Table 1. Seven patients were staged IIIB (FIGO staging), three IIB, two IB2, and one IVA. All the patients were treated between year 2018 and 2019.

**TABLE 1:** Patient characteristics

Patient	T	N	M	Age	Acute gastrointestinal tract (GI) Tox.	Acute genitourinary (GU) Tox.
1	3B	1	0	44	G0	G2 vaginal pain
2	3B	1	0	39	G1 diarrhea	G2 frequency, G1 dysuria
3	2B	X		35	G0	G2 vaginal pain, G3 vaginal hemorrhage
4	2B	X		58	G0	G0
5	2B	1	0	42	G2	G2 pelvic
6	2A2	1	0	37	G2 diarrhea, G1 fecal incontinence	G1 renal
7	3B	0	0	78	G0	G0
8	2B	0	0	48	G3 pelvic/lower abd.	G0
9	3B	1	0	52	G2 constipation	G2 vaginal hemorrhage
10	1B	X		26	G1 constipation	G1 frequency, G1 vaginal hemorrhage, G2 vaginal discharge
11	3B	0	0	49	G0	G1 vaginal hemorrhage, G2 dysuria
12	4A	0	0	63	G4 bleed	G1 vaginal hemorrhage, G2 dysuria
13	3B	X		41	G0	G3 vaginal stricture, G1 dysuria, G1 freq.

*Note*: G0 = Grade 0, G1 = Grade 1, G2 = Grade 2, G3 = Grade 3, and G4 = Grade 4.

### 2.2 Toxicity associated with brachytherapy

Most of the patients in our study had gastrointestinal tract (GI) and genitourinary (GU) issues prior to therapy. Some of the patients were suffering acute GI and GU toxicity within 3 months of brachytherapy completion. Most of the patients suffering from grade 0 (G0) to grade 3/4 (G3/G4) acute GI and GU toxicity. As shown in Table 1, some patients have shown a toxicity grade G0, when in reality they did experience toxicity that was not recorded. Vaginal hemorrhage is a common issue during brachytherapy with interstitial needles, so G1 vaginal hemorrhage could be expected for any patient in the acute setting.

### 2.3 Target volumes

HRCTV volumes for patients in this study were defined by the physician at the time of clinical treatment. Volumes started at the cervical os in the inferior aspect and are defined superior into the uterus, centered roughly around the applicator's tandem. The volumes were roughly uniform in thickness in the anterior/posterior direction, with a thickness between 3.5 and 5.7 cm. The volumes tended to be wider laterally in the superior aspect than close to the os, between 6.4 and 8.7 cm at the widest point. The difference between the left and right extensions of the target volume relative to the tandem was not greater than 1 cm for any fraction. For optimization, an HRCTV Opti that extends the HRCTV to include the area around the ring is used, though it does not alter the most extreme extensions of the target volume.

### 2.4 Treatment replanning

Twenty‐six single‐fraction original treatment plans from the Oncentra Brachytherapy planning system at Stephenson Cancer Center were used for dose evaluation with the Venezia applicator, where 2 plans had 16 needles and 24 plans had 12 needles in the applicator. The original plan's dose distributions, including all needles, were considered baseline for this study. To replan each original plan, each case of the patient was copied for modification. The replans were performed by removing a number of needle reconstructions from original plans within the system, so they would not be used for optimization. The Inverse Planning Simulated Annealing algorithm (Oncentra Brachy Planning, Elekta) was used to optimize the replans with the same optimization parameters used in original plans. Most plans were originally calculated with standardized optimization objectives.

### 2.5 Needle configurations

Nine new configurations have been designed for implementation in replans, specified in two ways in Table 2. Each configuration consisted of a different, reduced number of interstitial needles compared to the original treatment plans. The original plans included a full load of 12–16 needles, including both parallel and 30‐degree oblique needles. The applicator channels shown in Figure 1, where A, C, and E, G are oblique needle channels, and B, D, F, and H are parallel needle channels.

**TABLE 2:** Nine needle configurations

**Location of needles being removed**	**Number of needles removed**	**Name of needle configurations**
Most anterior two needles removed/1A and 2A removed	2	1A and 2A
Most posterior two needles removed/1F and 2F removed	2	1F and 2F
Most anterior and posterior four needles removed/1A, 2A, 1F and 2F removed	4	1 + 2 AF
Both sides oblique needles only removed/both sides A, C, E removed	6	BO
Both sides parallel needles only removed/both sides B, D, F removed	6	PO
Only left‐sided oblique needles removed/1A, 1C, 1E removed	3	OBL
Only right–sided oblique needles removed/2A, 2C, 2E removed	3	OBR
Only left‐sided parallel needles removed/1B, 1D, 1F removed	3	OPL
Only right parallel needles removed/2B, 2D, 2F removed	3	OPR

For each fraction, a new plan was generated for each configuration, and, in total, 234 replans from 26 fractions were produced.

### 2.6 Metrics of dose comparison

The dose comparisons and analysis between original and replans were performed based on the volumetric dose metrics and consideration during analysis. Comparisons included differences in percentage dose coverage to 90% of HRCTV *D*90, and percentage dose to 2 cm^3^ of the bladder, rectum, sigmoid, and bowel, which were derived from dose–volume histograms (DVHs). Within the Oncentra treatment planning system, the highest dose limit, sample point, and bins in the DVH setting were set to the highest values possible in the system. The values were dose limit = 16, sample point = 200000, and bins = 800.

The dose goal of this study mainly emphasized the dose coverage to the HRCTV *D*90, while trying to keep the dose to OAR within the dose constraints recommended to them.

The analysis of the replans was evaluated in a binary way, passing or not, based on specific criteria. A list of the passing criteria is included in Table 3. If a replan able to achieve dose coverage to target (HRCTV *D*90) and all critical organs according to department's standard criteria, or a limit set by this study, we called it a passed replan.

**TABLE 3:** Standard criteria for passing replans

Metrics	HRCTV *D*90	Bladder 2 cm^3^	Rectum 2 cm^3^	Sigmoid 2 cm^3^	Bowel 2 cm^3^
Institutional dose criteria (% of prescription dose)	≤100	<85	<65	<65	<65
Limit difference set by study (%)	≤−2	≤5	≤5	≤5	≤5%

Abbreviation: HRCTV, high‐risk clinical target volume.

This process was performed in two steps:

**Step 1—Evaluation of passing replans based on standard criteria**

The first step of this analysis evaluated what we considered “passing replans,” where new replans were labeled “passing” if they remained within all of our department's standard criteria, depending on the needle configurations. The standard recommended criteria for HRCTV *D*90 ≥ 100%, and for critical structures, *D*2 cm^3^ < 85% of prescription (Rx) dose (510 cGy) to bladder, <65% of Rx dose (390 cGy) to rectum, sigmoid, and bowel of prescribed dose.

An individual replan was also considered “passing,” even if it could not provide the same dose coverage as original plan but meet the department's standard recommended criteria for HRCTV *D*90 ≥ 100%. As the removal of the needles reduces the degree of freedom for dose optimization, so the replans often provided less coverage than the original. Similarly, for critical organs (OAR), replans were also called “passing” even if they could not provide the same dose sparing as the original plans but were able to achieve the department's standard recommended criteria for dose to those OAR.

For any replans based on original plans that themselves were not meeting departmental dosimetry criteria, the new replans were considered passing if the originally failing dose metrics were not substantially worse. We set the limit of relative dose difference between originals and replans to be ≤2% for the HRCTV *D*90 and ≤5% for critical organ *D*2 cm^3^ of OARs compared with original plans.

This dose comparison was performed for each new needle configuration for each of the fractions.

**Step 2—Overall percentage of passing replans**

The overall percentage of passing replans for each configuration was evaluated based on the combination of metrics. Replans were considered “failed” overall if any one dose comparison metric failed. Then, an overall percentage of passing replans were calculated with respect to each new configuration.

Additionally, plans were binned based solely on the total number of needles removed (two, three, four, and six needles), and the passing rates per group were analyzed.

## 3 RESULTS

Tables 4, 5, 6, 7, 8 indicate the average percentage of dose differences between the full‐loading and replans based on the nine needle configurations for HRCTV *D*90, *D*2 cm^3^ of bladder, rectum, sigmoid, bowel, respectively, including the mean, maximum, and minimum dose coverage. It also shows the percentage of the passing replans. Positive values indicate that the original plan's value is larger and negative values indicate that replan value is larger.

**TABLE 4:** High‐risk clinical target volume (HRCTV) *D*90 dose calculation for all configurations

**Name of needle configuration**	**Mean dose (cGy)**	**%Dose difference (mean)**	**Maximum dose (cGy)**	**Minimum dose (cGy)**	**Standard deviation**	**Plan passing rate (%)**
1A and 2A	651.6	−0.9	729.7	535.7	43.8	96.2
1F and 2F	639.6	0.9	734.5	527.6	49.3	84.6
1 + 2 AF	644.4	0.2	730.5	546.3	44.3	88.4
OBR	622.6	3.7	697.5	452.8	57.3	65.4
OPR	628.7	2.7	739.4	447.7	61	80.8
OBL	623.0	3.6	733.6	422.9	63.4	80.7
OPL	597.7	7.5	713.2	438.2	66.5	53.8
BO	573.2	11.4	712.6	283.6	93.2	50.0
PO	536.5	17.2	718.9	180.5	116	30.8

**TABLE 5:** Bladder 2 cm^3^ dose calculation for all configurations

**Name of needle configurations**	**Mean dose (cGy)**	**%Dose difference (mean)**	**Maximum dose (cGy)**	**Minimum dose (cGy)**	**Standard deviation**	**Plan passing rate (%)**
1A and 2A	487.5	−0.4	633.4	356.8	54.8	96.2
1F and 2F	492.2	−1.3	673.3	349.8	61.0	92.3
1 + 2 AF	491.0	−1.1	670.1	354.4	60.8	84.6
OBR	499.7	−3.1	637.2	380.8	56.5	76.9
OPR	498.5	−2.7	677.3	355.7	56.2	92.3
OBL	501.0	−3.2	693.2	347.4	63.5	80.8
OPL	493.8	−1.7	641.9	343.8	61.2	96.2
BO	513.0	−5.8	707.3	380.3	69.9	57.7
PO	514.5	−6.1	692.8	352.1	67.1	80.7

**TABLE 6:** Rectum 2 cm^3^ dose calculation for all configurations

**Name of needle configurations**	**Mean dose (cGy)**	**%Dose difference (mean)**	**Maximum dose (cGy)**	**Minimum dose (cGy)**	**Standard deviation**	**Plan passing rate (%)**
1A and 2A	405.0	−1.8	506.8	186.7	68.8	84.6
1F and 2F	403.8	−1.5	518.3	191.8	69.2	92.3
1 + 2 AF	405.2	−1.9	514.3	192.8	69.6	84.6
OBR	407.5	−2.7	502.4	210.9	68.4	76.9
OPR	412.4	−3.7	536.6	202.6	70.0	80.8
OBL	408.6	−2.7	509.1	195.5	70.1	80.8
OPL	411.4	−3.4	534.4	195.4	73.2	73.1
BO	412.7	−4.0	515.5	218.1	71.3	73.1
PO	427.4	−7.1	634.2	192.0	88.5	50.0

**TABLE 7:** Sigmoid 2 cm^3^ dose calculation for all configurations

**Name of needle configurations**	**Mean dose (cGy)**	**%Dose difference (mean)**	**Maximum dose (cGy)**	**Minimum dose (cGy)**	**Standard deviation**	**Plan passing rate (%)**
1A and 2A	428.5	−1.1	600.8	351.5	62.9	84.6
1F and 2F	426.9	−0.7	584.2	332.6	64.4	80.7
1 + 2 AF	428.4	−1.1	606.8	342.6	65.6	80.8
OBR	433.5	−2.4	553.7	325.1	67.6	65.4
OPR	448.7	−6.1	582.6	334.5	67.3	53.8
OBL	427.6	−1.0	572.1	341.1	63.8	76.9
OPL	439.3	−3.6	606.5	359.0	72.3	69.2
BO	430.7	−1.7	565.1	322.9	67.5	69.2
PO	440.0	−3.6	685.0	238.3	93.9	57.7

**TABLE 8:** Bowel 2 cm^3^ dose calculation for all configuration

Name of needle configurations	Mean dose (cGy)	%Dose difference (mean)	Maximum dose (cGy)	Minimum dose (cGy)	Standard deviation	Plan passing rate (%)
1A and 2A	297.3	−2.4	452.6	83.7	105.7	100.0
1F and 2F	298.9	−2.8	462.6	83.9	107.3	100.0
1 + 2 AF	297.3	−2.4	452.6	83.7	105.7	92.3
OBR	298.7	−2.5	489.2	79.4	107.6	96.2
OPR	302.3	−3.8	443.9	85.5	108.6	96.2
OBL	294.1	−1.5	450.1	83.2	103.9	100.0
OPL	289.1	1.0	528.3	82.8	111.6	92.3
BO	297.0	−2.2	484.1	80.8	106.3	96.2
PO	268.0	7.9	448.0	79.2	103.2	92.2

**FIGURE 3:**
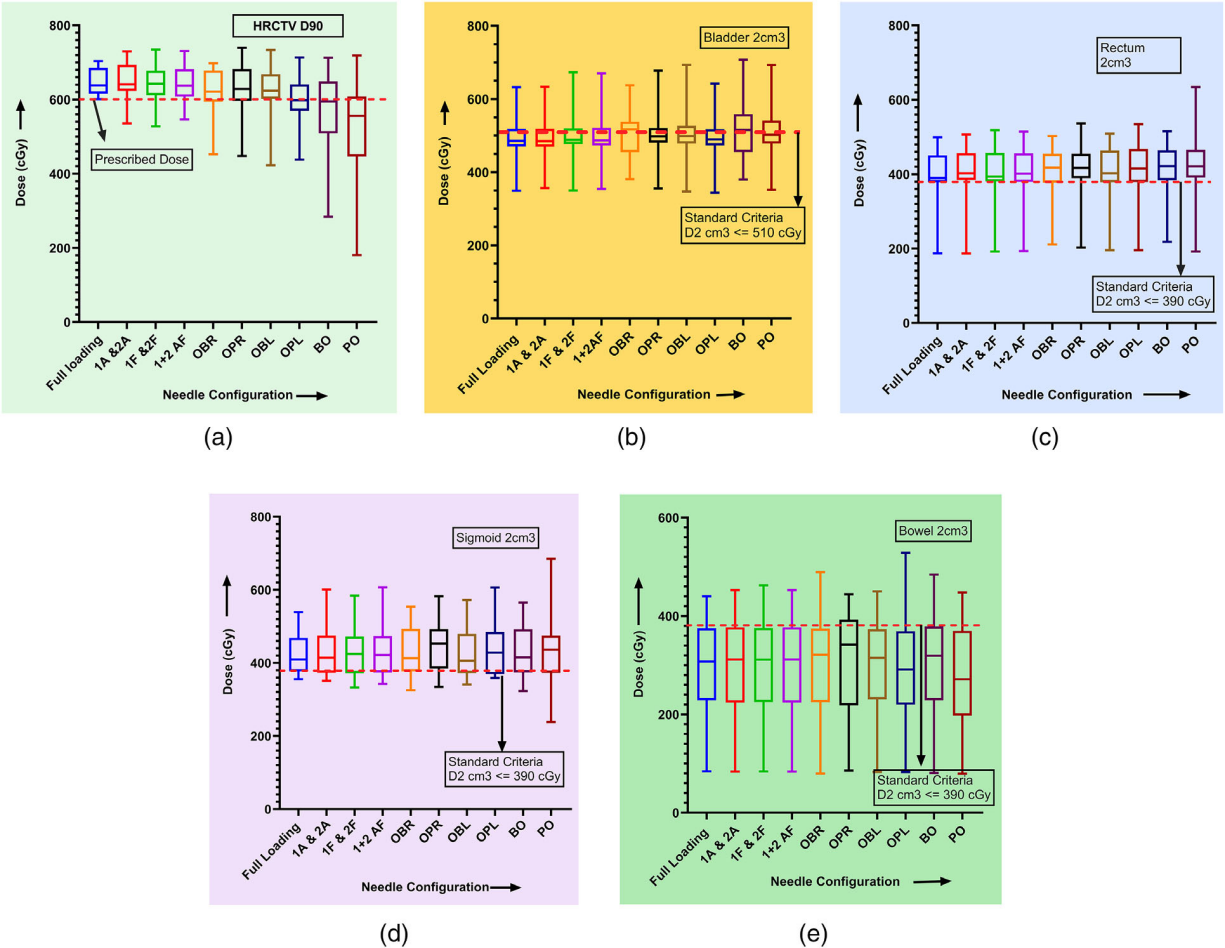
Box and whiskers plots displaying dose distribution for full‐loading and replan with different needle configurations in the (a) high‐risk clinical target volume (HRCTV) *D*90, *D*2 cm^3^ of (b) bladder, (c) rectum, (d) sigmoid, (e) bowel, respectively, where red dash line indicates the institutional standard dose criteria for each dose metrics. Each box contains mean, maximum, and minimum dose values. Lower, median, and upper quartiles of the box correspond to the 25%, 50%, and 75% of data distribution. The upper and lower whiskers represent data outside the middle 50%, that is, the lower 25% of data (minimum) and the upper 25% of data (maximum).

Figure 3 contains “box and whiskers” plots displaying dose comparison for originals and replans with different needle configurations, based on the HRCTV *D*90, *D*2 cm^3^ of bladder, rectum, sigmoid, and bowel related to the data included in Tables 4, 5, 6, 7, 8.

Table 9 indicates the overall percentage of passing replans based on the location of needles being removed. The removal of the most anterior and the most posterior needles separately or together showed the highest overall passing rate, where the removal of 1A and 2A provided 80.8% overall passing, the removal of 1F and 2F provided 61.50% overall passing. The removal of 1 + 2 AF provided 65.4% overall passing.

**TABLE 9:** Overall percentage of passing replans

**Needle configuration**	**Overall percentage (%) of passing replans**
1A and 2A	80.80
1 + 2 AF	65.40
1F and 2F	61.50
OBL	46.20
OPL	34.60
OBR	23.10
OPR	19.20
BO	19.20
PO	11.50

Removing all six parallel needles showed an 11.5% passing rate. Removing only right‐sided or only left‐sided parallel three needles separately showed 19.2% and 34.6% passing, respectively. Removing only left‐sided or only right‐sided oblique three needles showed 46.2% and 23.1% passing, respectively. Removing all six oblique needles showed a 19.2% passing rate.

Table 10 indicates overall passing replans based on the number of needles being removed with respect to the total number of replans. We have removed two, three, four, and six needles, among them the 2 and 4 number of needles removed provided a higher number of passing replans.

**TABLE 10:** Overall passing replans based on the number of needles being removed

**Number of needles removed**	2	3	4	6
**Total number of passing replans (total replans)**	40 (52)	36 (104)	18 (26)	10 (52)
**Percentage (%) of passing replans**	76.90	34.60	69.20	19.20

Table 11 shows the total number of replans failed with different needle configurations. It also includes the percentage of failing for each critical organ and the HRCTV *D*90. The sigmoid 2 cm^3^ provided the most failing replans for critical organs at 68, whereas the HRCTV provided the most failing replans overall at 73.

**TABLE 11:** Total number of replans failed with percentage of failing for each critical organ for different needle configurations

**Name of needle configuration**	**HRCTV *D*90**	**Bladder 2 cm^3^**	**Rectum 2 cm^3^**	**Sigmoid 2 cm^3^**	**Bowel 2 cm^3^**
1A and 2A	1	1	3	4	0
1 + 2 AF	3	4	3	5	0
1F and 2F	4	3	2	5	0
OBL	5	5	5	6	0
OPL	12	2	7	8	2
OBR	9	6	6	9	1
OPR	8	5	5	12	1
BO	13	11	7	8	1
PO	18	5	5	11	2
**Total no. of failed replans**	**73**	**42**	**43**	**68**	**7**
Failing percentage (%)	31.20	17.90	18.40	29.10	3

Table 12 indicates the number of original plans that failed to meet standard criteria for critical organs, and the percentage of replans derived from those failing original plans that passed the 5% difference criteria, with different needle configurations.

**TABLE 12:** The percentage of replans passed with original plans that failed to meet organ standard criteria

Organs	No. of original plans failed	1A and 2A (%)	1F and 2F (%)	1 + 2 AF (%)	OBR (%)	OPR (%)	OBL (%)	OPL (%)	BO (%)	PO (%)
Bladder	5	100	80	80	100	80	60	100	40	80
Rectum	11	91	100	82	82	82	73	73	64	45
Sigmoid	14	86	79	79	64	57	71	71	64	64
Bowel	4	100	100	100	75	100	100	75	75	75

There were several plans where the original plans had worse dose coverage than the department's standard recommended criteria. The number of original plans failing was 5, 11, 14, and 4 for bladder, rectum sigmoid, and bowel, respectively. Our new needle configurations provided a higher percentage of passing replans for those failed original plans, noting that in this case passing refers to plans which were not made substantially worse in target coverage or organ dose due to the removal of needles. The percentage of passing replans regarding failing original plans ranged from 1A and 2A = 86% to 100%, 1F and 2F = 79% to 100%, 1 + 2 AF = 79% to 100%, OBR = 64% to 100%, OPR = 57% to 100%, OBL = 60% to 100%, OPL = 71% to 100%, BO = 40% to 75%, PO = 45% to 80% to all critical organs. A lower number of passing replans came from BO and PO needle configurations.

## 4 DISCUSSION

The comparison between full‐loading original plans and replans with a reduced number of needle configurations did not show more than 0.9% mean dose difference in the HRCTV *D*90 dose coverage, with configuration 1A and 2A, 1F and 2F, or 1 + 2 AF. One general explanation is seen in the shape of the HRCTV planned to in this study. When HRCTV is larger than the standard distribution, it tends to be larger in the lateral and superior aspects, rather than anterior or posterior. These three configurations provided the highest percentage of the passing plans for HRCTV *D*90, 1A and 2A = 96.2%, 1F and 2F = 84.6%, and 1 + 2 AF = 88.4%, respectively.

This study found that the removal of the only left‐sided (OBL), or right‐sided (OBR), or all of the oblique needles (BO) provided mean dose difference between replans and full‐loading of 3.6%, 3.7%, and 11.4% for HRCTV *D*90, respectively. These configurations showed for a percentage passing rate of OBL = 80.7%, OBR = 65.4%, and BO = 50%, respectively, for the HRCTV *D*90. Configurations OPL, OPR, and PO showed a mean dose difference of 7.5%, 2.7%, and 17.2%, respectively. These configurations gave the percentage of passing plans, OPL = 53.8%, OPR = 80.8%, and PO = 30.8%, respectively, for the HRCTV *D*90. Configurations BO and PO provided mean HRCTV *D*90 dose 573.2 ± 93.2 and 536.5 ± 116.0 cGy, respectively. They did not even achieve institutional recommended dose coverage (600 cGy) as a higher number of needles (six needles) being removed. For BO or PO, there is a substantial reduction in the degrees of freedom for dose optimization, as both oblique and parallel needles have a strong contribution in dose coverage to the HRCTV *D*90 by reaching the target in both the parametrium and the vaginal extensions. It also indicates that both parallel and oblique needles are generally required, meaning the length of the tandem seems to be a limiting factor in these cases as well as the lateral extension of the tumor. At the most superior aspect of the target volumes, the oblique needles are simply too far from the center line to provide meaningful coverage. A recent study by Kissel et al.^17^ showed that parallel and oblique needles together versus only parallel needles substantially increased median *D*90 HRCTV and intermediate‐risk CTV: 85.9 Gy (range, 83.2–90.3 Gy) versus 81.5 Gy (range, 77.4–84 Gy) and 68.7 Gy (range, 66.3–72.3 Gy) versus 67 Gy (range, 64.3–69.1 Gy). The dose to OARs was not substantially different in this study. It indicates the combined contribution of the parallel and oblique needles together is important in dose coverage for abnormally shaped targets. Regarding OPL, OPR, OBL, and OBR plans, their poorer performance may be attributed to the laterally symmetrical nature of the target volumes in this study. With no differences in lateral extension from the tandem midline greater than 1 cm, it is clear for the cases studied that a symmetric needle pattern is ideal. We would recommend using symmetrical loading patters in all but the most exceptional cases.

The replans showed little variation in dose coverage over the full‐loading in terms of the critical organs sparing by this study. It had been found that the dose coverage to 2 cm^3^ of the bladder showed the little variation from the full‐loading for all new configurations except one, BO. Most of the configurations provided a percentage of passing replans ranging from 76.9% to 96.2%, where BO gave only 57.8%. There were only 42 (17.9%) replans with bladder 2 cm^3^ being failed among 234 replans from nine configurations to achieve the dose coverage compared to the full‐loading. A study by Bansal et al.^18^ showed that interstitial needles (number ranged from 18 to 26) combined with an intrauterine tandem resulted in significantly higher doses to 2 cm^3^ of the bladder than intracavitary alone (Fletcher tandem and ovoids), with comparable doses to 2 cm^3^ for the rectum and sigmoid. It supported our study to use a lesser number of needles to reduce the dose to the bladder 2 cm^3^, where they used 18–26 needles and this study tried to reduce the needles number to 6–10.

The 2‐cm^3^ coverage of rectum and sigmoid with replans provided by this study also showed the comparable coverage with the full‐loading, where the mean dose difference for all configurations was ranging from −1.5% to +7.9% for rectum 2 cm^3^ and −0.7% to −6.1% for sigmoid 2 cm^3^. The mean value for dose to 2 cm^3^ with full‐loading (original) for both rectum and sigmoid was above the institutional standard criteria (65% of prescribed dose or ⩽390 cGy). The number of failing replans for all replan configurations were 43 (18.4%) and 68 (29.1%) for rectum 2 cm^3^ and sigmoid 2 cm^3^, respectively. The original plans often failed because of a tendency of the sigmoid to wrap around the treatment area closely, in which cases it receives higher than desired amounts of radiation regardless of the number of needles.

The bowel showed great consistency with all replan configurations, with only 7 (2.9%) replans failing to meet the criteria. In most cases, the bowel is far enough superior to the treatment area to not be a great concern, especially with the bladder being full in all treatments. A study by Walter et al.,^16^ with only one to six needles per fraction, found the consistent dose to bladder 2 cm^3^, dose reduction to rectum 2 cm^3^ (58.7 Gy with needles vs. 62.5 Gy with intracavitary only), and improvement to HRCTV *D*90 (90.7 Gy with needles vs. 80.8 Gy with intracavitary only) with a Venezia applicator. Their study agreed with our finding of using 6–10 needles in combined interstitial and intracavitary brachytherapy, where our original plans had 12–16 needles.

This study also found that only two original plans required full‐loading of needles (all replans failed). Removal of 2 or 4 needles gave a higher number of passing plans, 40 (76.9%) and 18 (69.2%), respectively, including configurations 1A + 2A, 1F + 2F, and 1 + 2 AF. Removal of 3 or 6 needles showed 36 (34.6%) and 10 (19.2%) passing replans, respectively, including configurations PO, BO, OPL, OPR, OBL, and OBR.

When considering plans evaluated from originals with failing critical structure metrics, the poor performance of BO and PO configurations can be explained by the spreading of target size or volume abnormally toward oblique and parallel sides, bilaterally. When all oblique or parallel needles are removed, it could be difficult to cover the dose to these bilateral sides.

Several new needle configurations showed worse dosimetry for one of the critical organs with the reduced number of channels. This is most likely due to the reduced degrees of freedom forcing the optimizer to add more weight to less optimal dwell locations to maintain coverage of the HRCTV.

In general, the removal of needles had little effect on the overall dwell times of the plans. Revised plans saw gains and losses of less than 6% (<30 s with a source around 10 Ci) compared to the original dwell times. In practice, each channel that is not treated will save approximately 30 s of time from the actual treatment duration, due to fewer check cable runs and source travel time. Thus, we can deduce that the reduction of needles will yield at the worst no change in treatment time and, in the more extreme cases, reduce treatment times by 2–3 min.

The physician's expertise and understanding of the patient's anatomy and target shape were naturally important in creating each original plan. Each needle was not pushed into a specific depth but adjusted according to the physician's discretion. The depth of each needle in the applicator is usually not the same between among original plans, which depends mainly on the patient anatomy. The original plans used in this study had varied depth measurements relative to the applicator for all needle channels. This variation in depth leads to some inconsistency in the replan comparison as the new plans retained the same needle position inconsistency as the originals. However, each replan was only compared to its own original, so this inconsistency does not compromise the stated goal of this study.

Configuration 1 + 2 AF showed the best overall results in this study, which leaves two parallel and two oblique needles on each lateral side. It can be utilized to reach areas of bilateral extension, which is a relatively common phenomenon for the growth of cervical cancer, without increasing dose to the bladder and rectum from needles placed in close proximity. This suggests that laterally “mirrored” configurations are ideal when reducing the needle number. This idea is further validated by the lower number of overall passing replans with the removal of all right or left‐sided needles. A possible configuration with one parallel and one oblique needle on each side (four total needles) could be useful as this configuration would still have needles present on both lateral sides for both types of needles. Although we did not consider this configuration in our study, it would be a natural continued investigation of this trend.

A possible downside to the removal of the most anterior and posterior needles is the loss of their “anchoring effect” on the whole applicator, which adds stability to the placement during any patient transport and treatment. Another possible downside is the increase in dose heterogeneity caused by the limited dwell locations in an application. Historically, there have been varied philosophies for brachytherapy loading that favored dose homogeneity or heterogeneity in loading patterns for both high and low dose rate brachytherapy and should be discussed with the treating physician. Standard tandem and ring deliveries see HRCTV dose values at 300% or more in some parts of the target, depending on the applicator thickness and prescription point. It may be viewed as a feature: One study showed a 1.15–1.3 multiplying factor to biologically effective dose to the full target due to heterogeneity in brachytherapy plans.^19^ We would not consider the variation in heterogeneity among plans to necessarily be a problem unless it directly contributed to the escalation of organ dose to 2 cm^3^.

The use of advanced applicators like Venezia with additional channels increases the treatment process's complexity on several levels. This requires additional physician participation, treatment planning, physics, and treatment support personnel. Physicians should have the finest expertise to place the interstitial needles during the treatment. Treatment quality depends on proper reconstruction of needle channels and measurement of indexing lengths for channels used in treatment planning and physics. According to Wang et al.^20^ for treatment support personnel and physics, proper control, labeling, and attachment of multiple additional channels necessitate further dedication and organization.

## 5 CONCLUSIONS AND SUMMARY

The needle configurations with the removal of the most anterior and most posterior needles, whether separately or together, have shown comparably less variation with respect to the full‐loading original plans, for both HRCTV *D*90 and critical organs, and provided a higher number of passing replans. When the oblique and parallel needles were removed, whether separately or together, the dose coverage for HRCTV *D*90 was decreased compared to the full‐loading, as dose coverage of the target volume was dependent on both parallel and oblique needles, together. All replans had dose coverage within the defined tolerances, excepting two configurations due to the removal of necessary needle channels.

For critical organs, most of the new needle configurations neither provided improvement nor detriment to the organ doses, which was in‐line with the goal for this study.

The analysis of this study found that only two original plans among 26 required full‐loading, which indicates the feasibility of using the Venezia multichannel tandem and ring applicator to consistently produce dosimetrically comparable plans utilizing a fewer number of needle channels during combined intracavitary and interstitial HDR brachytherapy, potentially mitigating patient suffering and detrimental effects due to the use of a higher number of needles. Efforts should be made to seek a reduction in needles clinically, where feasible. In future studies, given these results, it may be possible to develop a guidance method for optimal needle placements based on target volumes defined on CT or MRI, potentially prior to the first applicator placement. A broader study, including more patients, variable needle placement depth, and more needle configurations, may also be necessary to inform such a guide.

## CONFLICT OF INTEREST

The authors have no Conflicts of Interest to disclose.
